# Predicting COVID-19 Using Retrospective Data: Impact of Obesity on Outcomes of Adult Patients With Viral Pneumonia

**DOI:** 10.7759/cureus.10291

**Published:** 2020-09-07

**Authors:** Hafeez Shaka, Sairam Raghavan, Valeria P Trelles-Garcia, Daniela Trelles-Garcia, Abdulrahman I Abusalim, Agata Parfieniuk, Pius E Ojemolon, Clark Azubuike

**Affiliations:** 1 Internal Medicine, John H. Stroger, Jr. Hospital of Cook County, Chicago, USA; 2 Internal Medicine, Saint Francis Hospital, Evanston, USA; 3 Anatomical Sciences, St. George's University, St. George's, GRD; 4 Emergency Medicine, University of Benin, Benin City, NGA

**Keywords:** obesity, mortality, covid-19, multi-viral pneumonia, pulmonary critical care

## Abstract

Background

Community-acquired pneumonia due to viral pathogens is an under-recognized cause of healthcare-associated mortality and morbidity worldwide. We aimed to compare mortality rates and outcome measures of disease severity in obese vs non-obese patients admitted with viral pneumonia.

Methods

Adult patients admitted with viral pneumonia were selected from the Nationwide Inpatient Sample of 2016 and 2017. The arms were stratified based on the presence of a secondary discharge diagnosis of obesity. The primary outcome was inpatient mortality. Secondary outcomes included sepsis, acute respiratory failure, acute respiratory distress syndrome, acute kidney injury, and pulmonary embolism.

Results and interpretation

In total, 89,650 patients admitted with viral pneumonia were analyzed, and 17% had obesity. There was no significant difference in mortality between obese and non-obese patients (aOR: 0.98, 95% CI: 0.705 - 1.362, p < 0.001). Compared to non-obese patients, obese patients had higher adjusted odds of developing acute hypoxic respiratory failure (aOR: 1.37, 95% CI: 1.255 - 1.513, p < 0.001), acute respiratory distress syndrome (aOR: 2.29, 95% CI: 1.554 - 3.381, p < 0.001), need for mechanical ventilation (aOR: 1.50, 95% CI: 1.236 - 1.819, p < 0.001), and pulmonary embolism (aOR: 1.69, 95% CI: 1.024 - 2.788, p = 0.040).

Conclusions

Obesity was not found to be an independent predictor of inpatient mortality in patients admitted with viral pneumonia. However, obesity is associated with worse clinical outcomes and disease severity as defined by the presence of complications, greater incidence of acute respiratory failure (ARF), acute respiratory distress syndrome (ARDS), need for mechanical ventilation, acute kidney injury (AKI), pulmonary embolism (PE), stroke, and sepsis.

## Introduction

Community-acquired pneumonia (CAP) is an important cause of mortality and morbidity across the world [1]. While traditionally attributed to be caused by bacterial pathogens, recent advances in diagnostic testing have identified viral pathogens in over 20% of patients with CAP. Viral and bacterial co-infection is associated with increased mortality, and the most common virus isolated is Influenza A [2, 3].

In December 2019, a novel coronavirus was identified to cause a viral pneumonia called Coronavirus Disease-2019 (COVID-19). This pneumonia was declared a pandemic by the World Health Organization (WHO) and has resulted in significant mortality worldwide. Obesity has been anecdotally reported to be strongly associated with worse outcomes for COVID-19 [4].

Obesity is a chronic disease which given its prevalence across the world, can be declared a pandemic in its own right. It is associated with impaired pulmonary function causing a restrictive physiology [5]. Obesity was also found to be an independent risk factor for worse outcomes in the 2009 H1N1 influenza pandemic [6-8]. This begs the question if obesity is associated with worse outcomes in viral pneumonia due to any virus. This could be from various mechanisms. It is possible that altered pulmonary physiology and the pro-inflammatory state seen in patients with obesity may be responsible for the worse outcomes in COVID-19 [9]. In this study, we aim to predict whether obesity is an independent risk factor for mortality and morbidity in viral pneumonia among adults, using population level data. This would provide a comparative model supporting possible risk factors involved in the current COVID-19 pandemic.

## Materials and methods

Design and data source

This was a retrospective study using the Nationwide Inpatient Sample (NIS) database for 2016 and 2017. The NIS is a database of hospital inpatient stays derived from billing data submitted by hospitals to statewide data organizations across the US, covering more than 97 percent of the U.S. population [10]. It approximates a 20-percent stratified sample of discharges from U.S. community hospitals, excluding rehabilitation and long-term acute care hospitals. This dataset is weighted to obtain national estimates [11].

Study population

The study population included adult patients who had a principal discharge diagnosis of viral pneumonia, including influenza due to identified novel influenza A virus with pneumonia (J09.X1), influenza due to other identified influenza virus with pneumonia (J10.0), influenza due to unidentified influenza virus with pneumonia (J11.0), and viral pneumonia, not elsewhere classified (J12). Patients were excluded if they had bacterial pneumonia. This cohort was further divided based on the presence of a secondary discharge diagnosis of obesity (E66.0, E66.1, E66.2, E66.8, E66.9, Z68.3, Z68.4). We combined both general codes for obesity as well as BMI specific codes to accurately capture obese patients.

Outcome measures

The primary outcome was inpatient mortality. Secondary outcomes included development of sepsis, septic shock, acute respiratory failure, acute respiratory distress syndrome, non-ST segment elevation myocardial infarction (NSTEMI), acute kidney failure (AKI), deep vein thrombosis (DVT), pulmonary embolism (PE), cerebrovascular accident, need for mechanical ventilation, vasopressors as well as mean length of hospitalization and mean total hospital charges.

Statistical analysis

The data analysis was done using Stata® Version 16 software (StataCorp, Texas, USA). Co-morbidities were calculated as proportions of the cohort and Chi-squared test was used to compare these characteristics between the subgroups. Multivariate regression analysis was done to adjust for possible confounders while calculating the primary and secondary outcomes. Confounders were obtained from literature review, and included variables from the validated MuLBSTA Score for viral pneumonia as well as the Pneumonia Severity Index (PSI) [12, 13]. A threshold p-value of <0.05 was set for statistical significance.

Ethical considerations

NIS databases lack patient, hospital or state level identifiers. This has enhanced patient protection and anonymity. This study was exempt from Institutional Review Board approval.

## Results

Patient characteristics

The combined NIS database for 2016 and 2017 contained over 71 million weighted hospital discharges of which 89,650 satisfied the inclusion criteria for the study. These patients were adults with a principal discharge diagnosis of viral pneumonia. Of these patients, 17.07% had a secondary diagnosis of obesity.

Table 1 details patient and hospital characteristics of the studied cohort. Relative to non-obese patients, obese patients were significantly younger (60.5 vs 69.5 years, p < 0.001), with a higher proportion of women (62.9 vs 53.4%, p < 0.001), Blacks (16.0 vs 10.5%, p < 0.001) and Hispanics (11.1 vs 9.6%, p < 0.001). Obese patients were more likely to have co-morbid hypertension (44.6 vs 40.6%, p < 0.001), diabetes (47.0 vs 28.4%, p < 0.001), history of past or current use of tobacco (40.1 vs 37.4%, p = 0.008) and chronic obstructive pulmonary disease (28.2 vs 25.2%, p < 0.001). Non-obese patients had a higher proportion of history of malignancy (39.4 vs 34.1%, p < 0.001).

**Table 1:** Patient and Hospital Characteristics of Hospitalizations for Viral Pneumonia by Obesity #: for 2017, CHF: Congestive heart failure, CKD: Chronic kidney disease, COPD: Chronic obstructive pulmonary disease, CVA: Cerebrovascular accident, IHD: Ischemic heart disease.

Variable	Overall	Non-obese %	Obese %	p-value
		n = 74,340 (82.9)	n = 15,310 (17.1)	
Patient characteristics				
Age, mean		69.5	60.5	<0.001
Women	55.1	53.4	62.9	
Racial distribution				<0.001
White	68.2	69.0	64.2	
Black	11.4	10.5	16.0	
Hispanic	9.9	9.6	11.1	
Others	10.5	10.9	8.7	
Insurance type				<0.001
Medicaid	66.9	69.4	54.7	
Medicare	10.0	9.2	13.7	
Private	20.1	18.7	27.1	
Uninsured	3.0	2.7	4.5	
Charlson Comorbidity Index score				<0.001
0	19.9	19.9	15.2	
1	27.4	27.4	25.8	
2	18.9	18.9	20.7	
≥3	33.8	33.8	38.4	
Median annual income in patient’s zip code, US$^#^				<0.001
1-43,999	28.6	28.0	31.5	
44,000-55,999	25.5	25.2	26.9	
56,000-73,999	24.5	24.7	23.6	
≥74,000	21.4	22.1	18.0	
Co-morbidities				
Diabetes	31.5	28.4	47.0	<0.001
Hypertension	41.3	40.6	44.6	<0.001
Smoking history	38.9	37.4	40.1	0.008
CHF	22.8	21.9	27.2	<0.001
CKD	16.8	16.6	18.0	0.058
Dyslipidemia	39.2	38.2	44.2	<0.001
Anemia	24.3	24.8	21.9	<0.001
Chronic IHD	23.8	24.2	22.1	0.015
Prior CVA	2.2	2.2	2.0	0.395
Malignancy	38.5	39.4	34.1	<0.001
COPD	25.7	25.2	28.2	<0.001
Oxygen dependent	6.7	6.1	9.2	<0.001
Hospital characteristics				
Hospital region				<0.001
Northeast	18.6	19.3	15.7	
Midwest	25.0	24.4	27.8	
South	35.3	35.1	36.3	
West	21.1	21.2	20.2	
Hospital bed size				0.022
Small	22.8	23.2	20.8	
Medium	27.8	27.6	28.5	
Large	49.4	49.2	50.7	
Urban location	87.7	87.4	89.2	0.013
Teaching hospital	62.3	61.9	64.2	0.025

Primary outcome: in-hospital mortality

The in-hospital mortality for patients principally admitted for viral pneumonia was 2.22% of the total cohort. There was no significant difference in the adjusted odds of inpatient mortality (aOR: 0.98, 95% CI: 0.705 - 1.362, p < 0.001) between obese and non-obese patients. We made adjustment for both hospital and patient variables including comorbidities.

Secondary outcomes

Compared to non-obese patients, obese patients had higher adjusted odds of developing acute hypoxic respiratory failure (aOR: 1.37, 95% CI: 1.255 - 1.513, p < 0.001), acute respiratory distress syndrome (aOR: 2.29, 95% CI: 1.554 - 3.381, p < 0.001), need for mechanical ventilation (aOR: 1.50, 95% CI: 1.236 - 1.819, p < 0.001), and pulmonary embolism (aOR: 1.69, 95% CI: 1.024 - 2.788, p = 0.040). Obese patients also had significantly longer length of hospitalization as well as higher total hospital charges relative to non-obese patients. Table 2 provides a summary of the clinical outcomes.

**Table 2:** Clinical Outcomes of Hospitalizations for Viral Pneumonia by Obesity *: Statistically significant, #: Adjusted mean difference, aOR: Adjusted odds ratio, CI: Confidence interval, ARDS: Acute respiratory distress syndrome, NSTEMI; Non-ST segment elevation myocardial infarction.

Outcome	Non-obese	Obese	aOR (95% CI)	p-value*
Primary outcome				
In-hospital mortality	2.3	1.7	0.98 (0.705 – 1.362)	0.905
Secondary outcomes				
Length of stay, mean	5.0	5.7	0.66^ # ^(0.420 – 0.898)	<0.001*
Total hospital charges, mean US$	44676	55,208	7994^# ^(3549 - 12,438)	<0.001*
Sepsis	3.0	4.1	1.28 (1.025 – 1.534)	0.029*
Septic shock	0.9	1.2	1.26 (0.839 – 1.887)	0.267
NSTEMI	1.9	1.6	0.99 (0.714 – 1.383)	0.971
Mechanically ventilated	3.4	5.9	1.50 (1.236 – 1.819)	<0.001*
Used pressors	2.4	1.7	0.84 (0.597 – 1.175)	0.304
Acute kidney failure	17.3	19.5	1.31 (1.171 – 1.461)	<0.001*
Acute respiratory failure	24.2	31.4	1.37 (1.255 – 1.513)	<0.001*
ARDS	0.6	1.6	2.29 (1.554 – 3.381)	<0.001*
Deep vein thrombosis	1.2	1.4	1.33 (0.945 – 1.858)	0.102
Pulmonary embolism	0.4	0.8	1.69 (1.024 – 2.788)	0.040*
Cerebrovascular accident	0.3	0.5	1.96 (1.040 – 3.678)	0.036*

## Discussion

In this study, we have shown that obesity is not an independent predictor for in-hospital mortality in patients admitted with viral pneumonia. However, obesity was found to be an independent predictor of complications of viral pneumonia, including acute respiratory failure (ARF), acute respiratory distress syndrome (ARDS), need for mechanical ventilation, acute kidney injury (AKI), pulmonary embolism (PE), stroke, and sepsis. Obesity was also found to be associated with a longer length of hospitalization and total healthcare expenditure in patients admitted with viral pneumonia.

Prior studies have attempted to evaluate the relationship between obesity and pneumonia with conflicting results. One systematic review concluded that obesity is associated with greater disease severity of pneumonia [14]. However, a meta-analysis of observational studies declared that while obesity is associated with increased incidence of pneumonia, it is associated with a lower mortality risk [15]. The variance with our study is likely due to heterogeneity in the studies especially in relation to etiology of pneumonia. This ambiguity in determining the relationship between obesity and outcomes in pneumonia is indicative of the complex role that weight plays in the physiology of the critically ill.

It is interesting to note that in our analysis, despite a greater incidence of serious complications from pneumonia, such as ARF, ARDS and sepsis, that obesity was not associated with greater inpatient mortality. It is possible that amongst non-obese patients, there were also included patients with protein energy malnutrition. This is supported by data from a pediatric population, which found that moderate-severe malnutrition was associated with greater mortality [16]. However, etiologies and characteristics of malnutrition between adults and children vary significantly. Adults are more likely to be malnourished from chronic medical conditions [17, 18].

Our study had the following limitations. First, as the NIS is an administrative database, and ICD-10 codes were used to confirm diagnoses rather than clinical parameters. Therefore, disease severity could not be adequately accounted for. To mitigate this, we adjusted variables for known severity indices using models from validated tools including the PSI. Secondly, it is possible that patients with clinic-radiological evidence of pneumonia and a negative microbiological workup, including viral PCR swabs were coded as “viral pneumonia not elsewhere classified (J12)”. This misclassification is also likely to be spread evenly across both the obese and non-obese patients making it an error rather than a bias. The lower proportion of obese patients in the cohort compared to the general population of US adult may point to obesity being under-coded in the NIS database [19]. Therefore, it is possible that obese patients were included in the non-obese arm for analysis, diluting our results. Finally, information regarding treatment regimens for patients is not reported in the database, and we could not account for this in our analysis.

We are however confident that the large sample size and relative homogeneity of the principal diagnosis makes this an important project that could have significant clinical implications.

## Conclusions

In summary, obesity is not an independent risk factor for mortality in adults with viral pneumonia. However, obesity is associated with increased complications in viral pneumonia with a greater incidence of ARF, ARDS, need for mechanical ventilation, AKI, PE, stroke, and sepsis. In addition, obese patients were found to have an increased healthcare utilization cost, measured by increased length of stay and total hospital charges. Hence, obese patients should be categorized as higher risk for these outcomes. This would inform preventative and early therapeutic measures to mitigate disease morbidity.
